# Validity and reliability of the Korean caregiver contribution to self-care chronic illness inventory

**DOI:** 10.1038/s41598-023-35084-w

**Published:** 2023-05-14

**Authors:** Juhee Lee, Eunyoung Kim, Misook Chung, Insun Yeom

**Affiliations:** 1 Mo-Im Kim Nursing Research Institute, Yonsei Evidence Based Nursing Centre of Korea: A JBI Affiliated Group, College of Nursing, Yonsei University, Seoul, Republic of Korea; 2 College of Nursing and Brain Korea 21 FOUR Project, Yonsei University, Seoul, Republic of Korea; 3 College of Nursing, University of Kentucky, Lexington, KY USA

**Keywords:** Health care, Signs and symptoms

## Abstract

The contribution of caregivers to self-care for chronically ill patients is important for improving patient outcomes. The Caregiver Contribution to Self-Care Chronic Illness Inventory (CC-SC-CII) has been used to assess caregivers’ contributions to three distinct aspects of self-care (maintenance, monitoring, and management) globally. This study aimed to examine the psychometrics of the Korean version of the CC-SC-CII with 230 family caregivers (mean age = 49.8 years, 70% women) of patients with chronic illness. We demonstrated that the CC-SC-CII-Korean has good reliability with acceptable internal consistency and construct validity for all three factors using confirmatory factor analysis. The CC-SC-CII-Korean is a reliable and valid instrument to measure the contributions of Korean caregivers to the self-care of patients with chronic illnesses.

## Introduction

The prevalence of chronic disease continues to increase due to population aging, healthcare technology gains, and life expectancy increases. According to the World Health Organization, chronic diseases account for about 71% of deaths worldwide, with cardiovascular disease, cancer, chronic respiratory disease, and diabetes having the highest mortality rates^1^. National healthcare expenses due to chronic diseases have been reported to account for more than 80% of all healthcare expenses, and the socioeconomic burden is expected to continue to increase^2^. One out of every three people in Korea has a chronic disease, and the proportion of healthcare expenses spent on chronic disease management out of total healthcare expenses rose from 72.5% in 2018 to 85.0% in 2020. In addition, seven of the top 10 causes of death as of 2021 were chronic diseases, and deaths due to chronic diseases accounted for 79.6% of all deaths^3^. Accordingly, it is necessary to efficiently respond to the burden caused by chronic diseases, including the financial burden of health insurance.

Most chronic diseases are incurable, and since their prognosis tends to be poor, they can negatively affect not only patients’ physical condition but also psychosocial aspects of their lives^4^. Among chronic diseases which affect pediatric patients, lifelong self-care is required through diet, exercise, stress control, insulin treatment, hormone treatment, or regular medical treatment, depending on the disease. Unlike adults with chronic illness, while going through puberty with a chronic disease, pediatric patients experience many psychosocial conflicts, and these psychosocial conflicts make self-care difficult. In addition, if self-care does not go well, a vicious cycle in which psychosocial conflicts intensify repeats^5^.

Patients with chronic illnesses perform daily self-care tasks to reduce the effects of their disease and manage symptoms^6^. In line with the mid-range theory of chronic disease self-care, self-care involves three key concepts: 1) self-care maintenance to uphold physical and emotional stability, 2) self-care monitoring to track behavior and watch for signs and symptom changes, and 3) self-care management to take appropriate action when signs and symptoms appear^7^. Since patients with chronic illness commonly experience difficulties with daily living, cognitive decline, and other complexities from long-term chronic illness^8^, they often depend on family and informal caregivers to help with disease management^9^. The scope of this care ranges from simple protective activities in daily life to complex and holistic activities to support not only physical help and survival, but also social, emotional, physical, and economic quality of life^10^. In particular, family caregivers provide emotional support and actively support chronic disease management based on the patient’s healthcare plan. Family caregivers help patients perform appropriate self-care. They help patients’symptom managenent and medication side effects^11^.

The contributions of caregivers to self-care for patients with chronic illnesses have been described as facilitating monitoring and recommending actions to patients’ clinical symptoms^12^. Family caregivers, who are generally informal caregivers, perform activities critical to the well-being and survival of patients with chronic diseases^13^. They support symptom and medication management, activities of daily life, and emotional and physical health^14^. Family caregivers play a significant role in the self-care of chronically ill patients, including giving medications, encouraging healthy behaviors, and monitoring and coping with symptoms. Such caregiver contributions may improve patient outcomes^15^. A recent dyadic intervention program for chronically ill patients and family caregivers increased the family caregiver's contribution to patients' self-care, resulting in physical and psychological improvements for the patient^16^. Caregivers’ emotional support for patients’ self-care resulted in reducing the number of emergency room visits and mitigating worsening symptoms^17^. As the contributions of caregivers are very important to the clinical outcomes of patients with chronic disease^18,19^, a comprehensive understanding of the treatment process for chronic diseases is needed through reliable scales that enable measurement of caregivers' contributions to self-care of chronically ill patients.

A few measures for evaluating the caregiver's contribution to self-care in chronic illness patients have been developed^20–23^. Among them, the Caregiver Contribution to Self-Care of Chronic Illness Inventory (CC-SC-CII) is one of the most widely used for measuring the contribution and efficacy of caregivers^23^. The CC-SC-CII allows the assessment of the contribution of caregivers to the self-care of chronically ill patients. As reported, CC-SC-CII was developed based on the Self-Care of Chronic Illness Inventory (SC-CII)^23^. This instrument has been used in Chinese and English after its reliability and validity were examined^24^. However, it has not been used in Korea. Unfortunately, there is no instrument to directly measure the contribution of the Korean caregiver to the self-care of patients with chronic illness. Although several instruments, including the Family Support Instrument^25^, the Korean Revised Caregiving Appraisal Scale^26^, and the Instrument for Measuring the Care Satisfaction of Home Health Nursing^27^, assess similar concepts of caregivers’ contribution, these scales were mainly developed to measure the level of support and satisfaction of family caregivers for patients. In other words, those scales do not measure caregivers’ contribution to the self-care performance of chronic disease patients since they evaluate the degree of communication, emotional, economic, and physical support of families with a chronically ill member or examine the degree of nursing satisfaction of caregivers. Based on previous studies that measured contributions of caregivers to the self-care performance of chronic disease patients^20–24^, it is necessary to develop a scale to understand the process of self-care in Korea. Therefore, the primary aim of this study was to verify the validity and reliability of CC-SC-CII in Korea and with the Korean language. The other aims were to contribute to understanding the caregiving process by providing a one-to-one perspective when measuring the self-care performance and contribution of chronic disease patients and to help healthcare professionals periodically evaluate the degree of contribution to chronic disease patients’ self-care performance.

## Results

### Characteristics of participants

Of the 230 caregivers (Table 1), most were women (70%) and married (80%). The mean age was 49.8 years (SD = 15.7). More than half of caregivers were college-educated (56%), employed (67%), and living with the patient (60%). Caregivers were spouses (38%), children (34%), or parents of patients (26%) with chronic illnesses. Caregivers provided an average of 22.6 h of care per week and had an average of 5.3 years of care. Caregivers took care of patients with chronic illnesses, including cardiovascular disease, cancer, diabetes mellitus, chronic obstructive pulmonary disease, hypertension, renal failure, and other chronic illnesses. Care recipients had an average of 2.9 illnesses, and their mean age was 63.2 ± 14.5 years (Table 1).

**Table 1:** Characteristics of caregivers and patients.

Characteristics	Categories	Caregivers	Patients
		M ± SD [Min–Max] or n(%)	M ± SD [Min–Max] or n(%)
Age	Years	49.75 ± 15.68 [22–70]	63.22 ± 14.51 [18–82]
Gender	Female	162 (70.4)	139 (60.4)
	Male	68 (29.6)	91 (39.6)
Marital status	Unmarried	45 (19.6)	–
	Married	185 (80.4)	–
Education	Elementary	7 (3.0)	19 (8.2)
	Middle	4 (1.7)	28 (12.2)
	High	54 (23.5)	112 (48.7)
	College	129 (56.1)	61 (26.5)
	Graduate	36 (15.7)	10 (4.3)
Employment status	Yes	155 (67.4)	–
	No	75 (32.6)	–
Income (× 10,000 KRW/monthly)	< 199	77 (33.5)	–
	200 ~ 299	38 (16.5)	–
	300 <	115 (50)	–
Relationship with patient	Spouse	88 (38.3)	–
	Child	79 (34.3)	–
	Parents	59 (25.7)	–
	Sister or brother	4 (1.7)	–
Living with patient	Yes	139 (60.4)	–
	No	91 (39.6)	–
Caregiving hours per week	Hour	22.63 ± 20.01 [5–58]	–
Period of caregiving (years)	Year	5.30 ± 5.91 [2–12]	–
Number of chronic illness	–	–	2.94 ± 1.45 [2–5]

*M* Mean, *SD* Standard deviation.

### Translation/back-translation

A translation/back-translation process was applied to the original scale, i.e., the CC-SC-CII^28^. In the Korean version of the CC-SC-CII (CC-SC-CII-Korean), a few words in the original CC-SC-CII were revised to fit Korean linguistics. For example, “Avoid tobacco smoke” in item #8 was modified to “Smoking-cessation” because it is a more familiar and culturally accepted word in Korean. Since a national chronic illness management project was in progress in Korea in 2007, the word smoking-cessation education was implemented in counseling by integrating similar words, including avoid tobacco smoke^29^. “Call the healthcare provider for guidance?” in item #19 was back-translated to “Ask the healthcare provider for guidance?” to reflect the Korean health care system. Visiting clinics without reservation is common and the easiest way to access the health care system in Korea^30^. Thus, asking for direct face-to-face guidance during clinic visits is a common approach rather than calling the medical staff.

### Item analysis

A 19-item CC-SC-CII instrument specifically measures caregivers' contributions to self-care maintenance, monitoring, and management of patients with chronic illness^23^*.* The average score for each item ranged from 2.92 to 4.40 points, with a maximum score of 5. Skewness ranged from − 1.66 to − 0.29, and kurtosis ranged from − 0.67 to 2.17, indicating that all items had a normal distribution. The corrected item / total correlation coefficient between individual items and all items was 0.30 or higher, confirming the internal consistency of the items (Table 2)^31^.

**Table 2:** Item analysis of the Korean version of the CC-SC-CII.

Items	M ± SD	Skewness	Kurtosis	ITC(r)
1	3.87 ± 0.98	− 0.84	0.69	0.59
2	4.22 ± 0.84	− 0.96	0.64	0.67
3	3.79 ± 1.07	− 0.63	− 0.24	0.42
4	3.58 ± 1.03	− 0.29	− 0.41	0.51
5	4.18 ± 0.93	− 1.10	0.88	0.67
6	4.40 ± 0.90	− 1.66	0.28	0.67
8	4.11 ± 1.37	− 1.31	− 0.19	0.40
9	4.19 ± 0.79	− 0.62	− 0.33	0.81
10	3.99 ± 1.00	− 0.92	0.31	0.74
11	4.24 ± 0.84	− 1.06	1.15	0.84
12	4.08 ± 0.88	− 0.82	0.45	0.78
13	4.26 ± 0.74	− 0.84	0.55	0.86
14	2.92 ± 1.23	− 0.52	− 0.35	0.41
15	3.87 ± 0.95	− 0.73	0.25	0.85
16	3.88 ± 0.93	− 0.58	0.02	0.79
17	4.05 ± 0.92	− 1.07	1.44	0.61
18	4.40 ± 0.72	− 1.25	2.17	0.71
19	4.01 ± 0.95	− 0.83	0.24	0.71
20	3.10 ± 1.25	− 0.52	− 0.28	0.44

*ITC* Item-total correlation, *M* Mean, *SD* Standard deviation, *CC-SC-CII-Korean* Korean version of the caregiver contribution to self-care of chronic illness inventory.

### Content validity

Four experts verified the content validity of CC-SC-CII-Korean. Polit and Beck^32^ recommend an appropriate content validity index of 0.90 or higher when 4 experts participate. All 19 items had item-content validity index (I-CVI) values of 1.0 and were therefore included as final items as they satisfied the criterion of 0.9 or higher^32^.

### Construct validity

**Figure 1:**
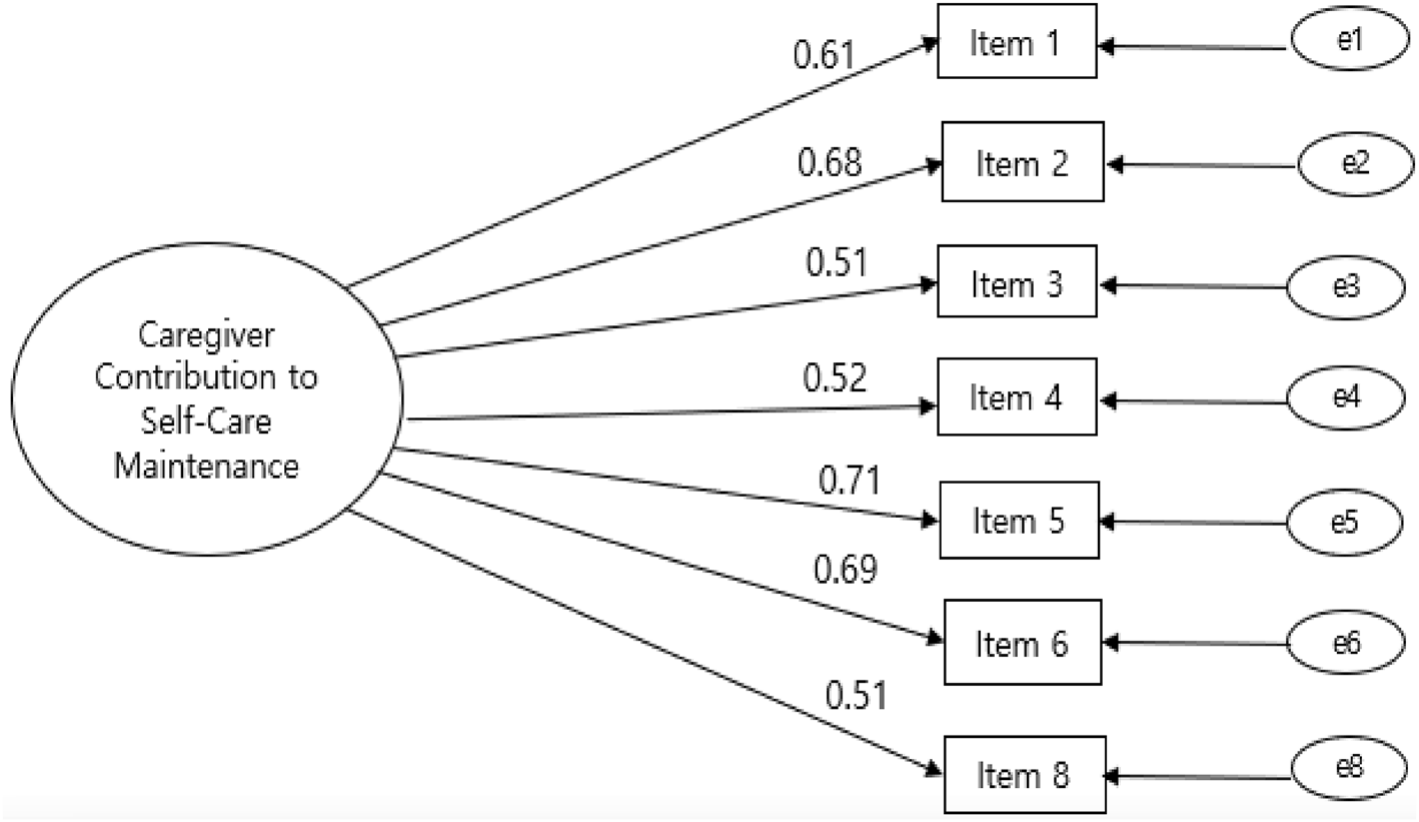
Confirmatory factor analysis of the Korean version of the Caregiver Contribution to Self-Care Inventory-Maintenance subscale. Note:  = Factor; = Factor loading; = Items;  = Error variance; Item 1 = enough sleep; Item 2 = avoid getting sick; Item 3 = physical activity; Item 4 = eat special foods or avoid certain foods; Item 5 = keep appointment for health care; Item 6 = take prescribed medicines; Item 8 = smoking-cessation.

**Figure 2:**
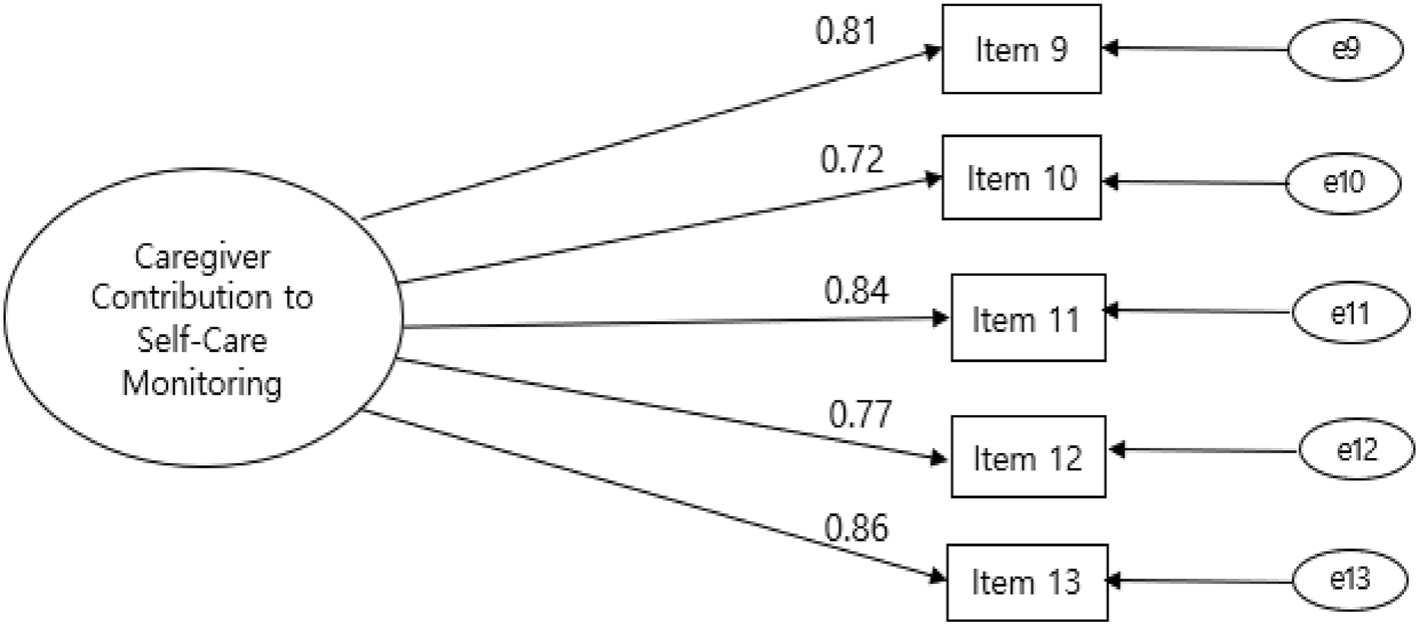
Confirmatory factor analysis of the Korean version of the Caregiver Contribution to Self-Care Inventory-Monitoring subscale. Note:  = Factor;  = Factor loading; = Items;  = Error variance; Item 9 = monitor the health condition; Item 10 = monitor for medication side-effects; Item 11 = monitor to changes in how one feels; Item 12 = monitor one tires more than usual; Item 13 = monitor for symptoms.

**Figure 3:**
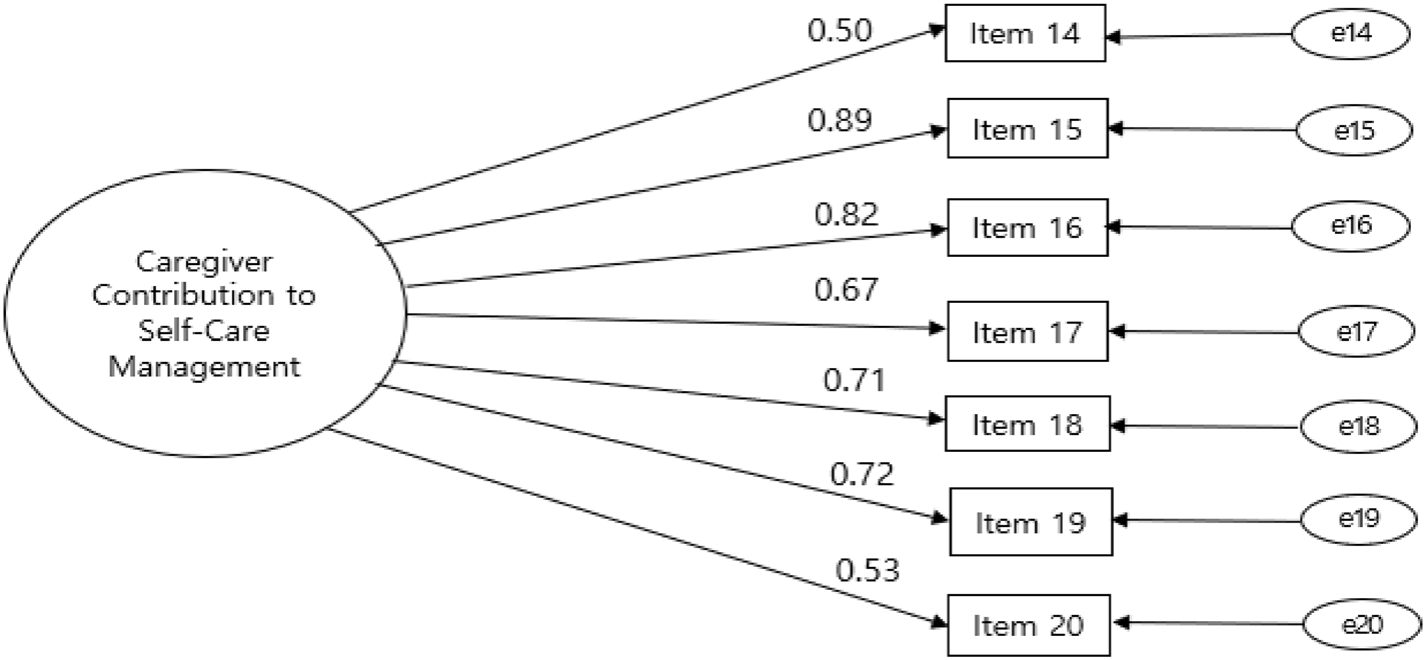
Confirmatory factor analysis of the Korean version of the Caregiver Contribution to Self-Care Inventory-Management subscale. Note:   = Factor;  = Factor loading;  = Items; = Error variance; Item 14 = recognize it as a symptom; Item 15 = care for eats or drinks; Item 16 = care for to change the activity level; Item 17 = care for to take a medicine; Item 18 = talk about the symptom to the healthcare; Item 19 = ask the healthcare provider for guidance; Item 20 = make him/her feel better.

**Figure 4:**
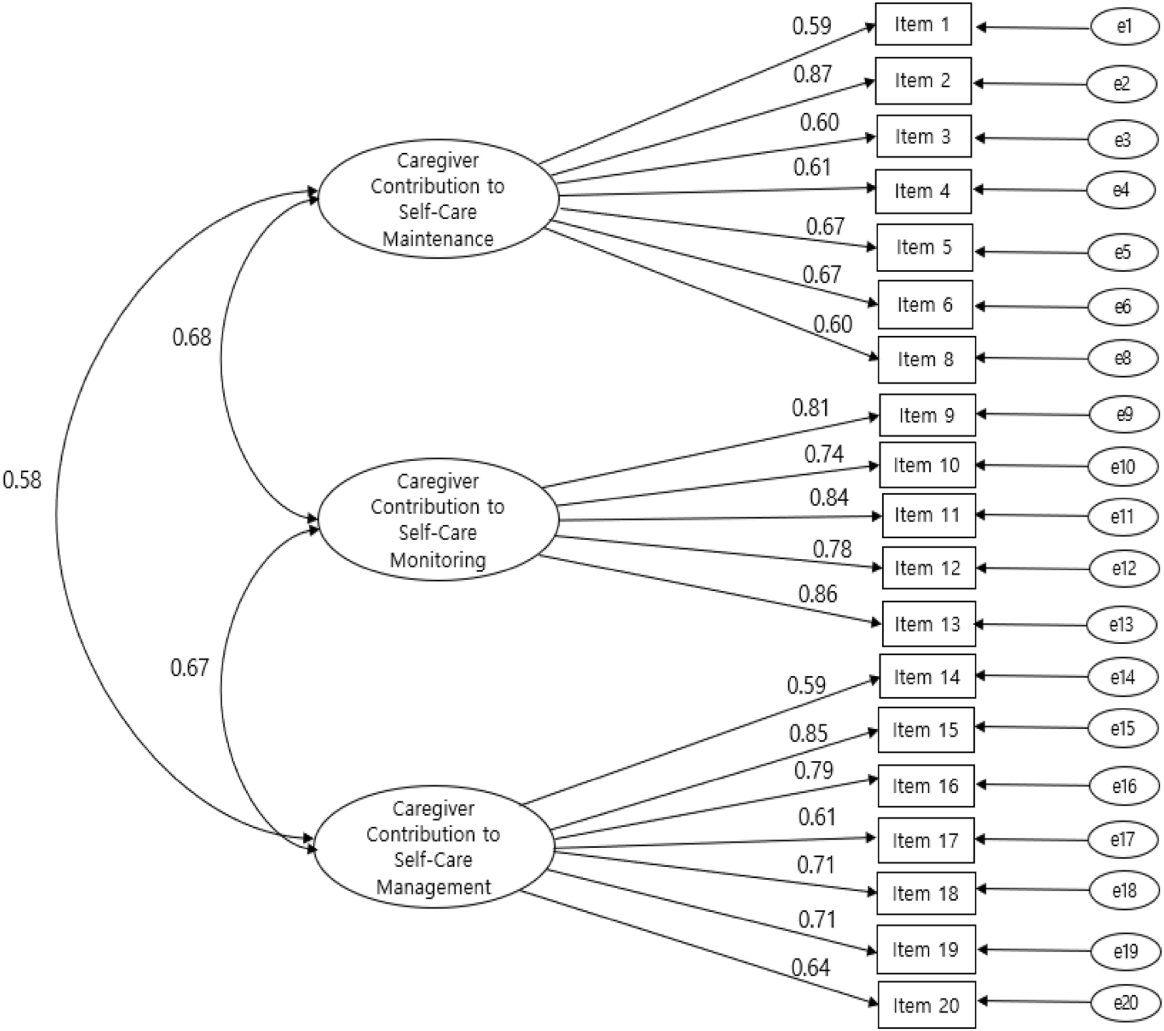
*Simultaneous* confirmatory factor analysis of the Korean version of the CC-SC-CII. Note:  = Factor correlation;  = Factor;  = Factor loading;  = Items; = Error variance; Item 1 = enough sleep; Item 2 = avoid getting sick; Item 3 = physical activity; Item 4 = eat special foods or avoid certain foods; Item 5 = keep appointment for health care; Item 6 = take prescribed medicines; Item 8 = smoking-cessation; Item 9 = monitor the health condition; Item 10 = monitor for medication side-effects; Item 11 = monitor to changes in how one feels; Item 12 = monitor one tires more than usual; Item 13 = monitor for symptoms; Item 14 = recognize it as a symptom; Item 15 = care for eats or drinks; Item 16 = care for to change the activity level; Item 17 = care for to take a medicine; Item 18 = talk about the symptom to the healthcare; Item 19 = ask the healthcare provider for guidance; Item 20 = make him/her feel better.

The confirmatory factor analysis (CFA) results for each of the three factors Caregiver Contribution to Self-care scales and index are presented below and in Figs. 1, 2, 3 and 4.

#### Confirmatory factor analysis

##### Caregiver contribution to self-care maintenance

In this study, the caregiver contribution to self-care maintenance was assumed to have the same two dimensions as in the study of the original instrument: health-promoting behaviors and illness-related behaviors. However, this model was not selected because the RMSEA value was over 0.08 (results were χ2 = 23.81, df = 15, *P* = 0.053; CFI = 0.89, TLI = 0.83, GFI = 0.76, AGFI = 0.75, RMSEA = 0.09, NFI = 0.82 and IFI = 0.81).

Instead, a model with a single-factor solution that yielded a supportive fit was identified. The seven self-care maintenance items showed suitable threshold levels (χ^2^ = 24.35, df = 14, *P* = 0.042) and a reasonable fit of the model index to the data (CFI = 0.97, TLI = 0.93, GFI = 0.97, AGFI = 0.87, RMSEA = 0.06, NFI = 0.93 and IFI = 0.97).

Squared multiple correlations ranged from 0.44 to 0.84, and the standardized factor loadings ranged from 0.51 to 0.71. The results of CFA in the self-care maintenance model supports that each set of items represents a single subjacent construct (Fig. 1).

##### Caregiver contribution to self‑care monitoring

In this study, the caregiver contribution to self-care monitoring was assumed to be the same dimension as in the original scale—that is, self-care monitoring behaviors. The analysis suggested a suitable model with a single-factor solution, as in the original scale. The five self-care monitoring items showed suitable threshold levels (χ^2^ = 90.35, df = 14, *P* < 0.001), and a reasonable fit of the model index to the data (CFI = 0.99, TLI = 0.99, GFI = 0.99, AGFI = 0.91, RMSEA = 0.03, NFI = 0.97, and IFI = 0.99).

The squared multiple correlations ranged from 0.74 to 0.87, and the standardized factor loading ranged from 0.72 to 0.86. The CFA of the self-care monitoring model offers evidence for acceptable fit (Fig. 2).

##### Caregiver contribution to self‑care management

The caregiver contribution to self-care management was assumed to have two secondary factors: autonomous behaviors and consulting behaviors. However, the results of the CFA did not fit this assumption well since the RMSEA was high (χ^2^ = 99.03, *P* = 0.04, df = 53; CFI = 0.87, TLI = 0.83, GFI = 0.85, AGFI = 0.81, RMSEA = 0.10, NFI = 0.81, and IFI = 0.84). Instead, the analysis indicated a more suitable model with a single-factor solution. The seven items for the caregiver contribution to self‑care management showed suitable threshold levels (χ^2^ = 108.80, *P* < 0.001, df = 51), and a reasonable fit of the model index to the data (CFI = 0.91, TLI = 0.93, GFI = 0.91, AGFI = 0.86, RMSEA = 0.07, NFI = 0.90, and IFI = 0.91).

The squared multiple correlations ranged from 0.59 to 0.85, and the standardized factor loading ranged from 0.50 to 0.89. The CFA of the self-care management model also supports offers evidence of an acceptable fit (Fig. 3).

##### Simultaneous confirmatory factor analysis of the CC-SC-CII-Korean

Figure 4 presents confirmatory factor analysis results to verify a general model with all items and a three-factor structure of the CC-CS-CII-Korean.

In the fit test for the 19-item CC-CS-CII-K model consisting of three factors, all fitness indices met the criteria (χ^2^ = 310.85 [*P* < 0.001], df = 149, CFI = 0.91, TLI = 0.91, NC = 2.09, RMSEA = 0.07, AGFI = 0.90, NFI = 0.8)^33^. The CFA showed that all factor loadings were significant, from 0.59 to 0.87 (Fig. 4). These results establish that the factors essential to the scales emerged plainly, even when the analysis was done on a mixture of items and factors.

Table 3 shows the test results of the construct validity of the CC-SC-CII-Korean items adopted through confirmatory factor analysis. In this study, construct validity of the items was confirmed based on three criteria. First, the standardized estimate (β) value was found to be 0.50–0.86, satisfying the standard criterion of 0.50 or more. Second, average variance extracted (AVE) the value obtained by dividing the sum of the provided values of the standardized load by the sum of the square of the standardized factor load and the sum of the study error variance was 0.54–0.72, which also satisfied the standard criterion of more than 0.50. Third, construct reliability (CR) the value obtained by dividing the square of the sum of the standardized factor loads by the square of the sum of the standardized factor loads and the sum of the study error variance was found to be above the standard value of 0.70 (range, 0.82–0.93) (Table 3), thus confirming the construct validity of the instrument by satisfying all three conditions.

**Table 3:** Parameter estimates, and item analysis in confirmatory factor analysis of the CC-SC-CII-Korean.

Domain (factor)	Item no	Non- Standardized estimate	Standardized estimate (β)	Squared multiple correlation	SE	C.R	*P*	M ± SD	AVE	CR	Cronbach’s α
Caregiver Contribution to Self-Care (CC-SC) Maintenance (Factor 1)	1	1.00	0.59	0.68	–	–	–	3.87 ± 0.98	0.72	0.93	0.75
	2	0.98	0.67	0.72	0.11	7.48	< 0.001	4.22 ± 0.84			
	3	0.77	0.52	0.44	0.15	5.21	< 0.001	3.79 ± 1.07			
	4	0.91	0.51	0.48	0.15	6.15	< 0.001	3.58 ± 1.03			
	5	1.09	0.67	0.84	0.15	7.49	< 0.001	4.18 ± 0.93			
	6	1.05	0.67	0.81	0.14	7.49	< 0.001	4.4. ± 0.89			
	8	0.97	0.50	0.45	0.19	5.09	< 0.001	4.11 ± 1.37			
Caregiver Contribution to Self-Care (CC-SC) Monitoring (Factor 2)	9	1.00	0.81	0.80	–	–	–	4.19 ± 0.78	0.54	0.82	0.90
	10	1.17	0.74	0.74	0.09	12.38	< 0.001	3.99 ± 1.00			
	11	1.10	0.84	0.84	0.08	14.62	< 0.001	4.24 ± 0.84			
	12	1.08	0.78	0.79	0.08	13.23	< 0.001	4.08 ± 0.88			
	13	0.99	0.86	0.87	0.07	14.97	< 0.001	4.26 ± 0.74			
Caregiver Contribution to Self-Care (CC-SC) Management (Factor 3)	14	1.00	0.50	0.59	–	–	–	2.92 ± 1.22	0.60	0.91	0.81
	15	1.61	0.85	0.85	0.27	6.04	< 0.001	3.87 ± 0.95			
	16	1.47	0.79	0.79	0.25	5.94	< 0.001	3.88 ± 0.92			
	17	1.13	0.61	0.66	0.21	5.47	< 0.001	4.05 ± 0.92			
	18	1.03	0.71	0.69	0.18	5.75	< 0.001	4.40 ± 0.72			
	19	1.38	0.71	0.72	0.24	5.76	< 0.001	4.01 ± 0.96			
	20	1.09	0.54	0.65	0.24	4.65	< 0.001	3.10 ± 1.25			

*AVE* Average variance extracted, *C.R.* Critical ratio, *CR* Construct reliability, *SE* Standard error, *M* Mean, *SD* Standard deviation.

The construct validity of the CC-SC-CII-Korean was also supported by the convergent validity. With reference to the Caregiver Self-Efficacy in Contributing to Patients Self-Care (CSE-CSC) scale^34^, the Pearson correlation coefficients for each of the three factors—caregiver contributions to self-care maintenance (r = 0.52, *P* < 0.001), caregiver contributions to monitoring (r = 0.55, *P* < 0.001), and caregiver contributions to management (r = 0.68, *P* < 0.001)—demonstrated the construct validity.

### Internal consistency reliability

An analysis of the internal consistency reliability and score distribution for the 19 items of CC-SC-CII-Korean, showed a Cronbach's α value of 0.75 for the seven items of the maintenance, 0.90 for the five items of the monitoring scale, and 0.81 for the seven items of the management scale (Table 3)^35^.

## Discussion

This study verified the validity and reliability of the CC-SC-CII-Korean which was developed to measure the contributions of caregivers to self-care for chronically ill patients.

Participants in this study differed from participants of the previous study in that parents of patients with chronic diseases are included. The average age of the patients in this study was 63.22 years (ranging from 18 to 82 years), and compared to the participants in the previous study (average age 76.6, ranging from 65 to 93 years), younger patients were included^23^. Nevertheless, the CC-SC-CII-Korean showed good validity and reliability among Korean caregivers for patients with chronic diseases. In the case of patients diagnosed with chronic diseases in childhood and adolescence, they perform self-care activities for the disease with support from their parents until young adults^36^. In other words, parents play an important role in caring for children with chronic diseases to adapt to the disease and maintain self-care behavior^36–38^. The results of this study confirmed the caregiver's contribution scale to the self-care behavior of chronic diseases applies not only to middle-aged and old patients but also to young adult patients. However, it needs to be further validated in a larger sample and wider age range of care recipients in the future.

Securing equivalence with the original instrument is important in instrument translation for cross-cultural healthcare studies. The translation/back-translation process alone cannot guarantee translation equality^28,39^. A committee went through the translation verification process for instructions, items, and response formats of the reverse translation instrument to increase the validity of the evaluation scale. The role of committee members was to review translation inconsistencies, inappropriate expressions, and concepts that may arise during the research process and revise each phrase as required to fit the target culture and sentiment^28^. As a result, two items were modified to more familiar expressions among similar words to suit the Korean culture and healthcare environment. This was also implemented in previous studies that evaluated the understandability of the original text and addressed any inaccuracies or ambiguities in the content of the translated scale relative to the original text^24^.

The CFA was used for the validation of structural feasibility in construct validity. Results showed that the CC-SC-CII-Korean had three valid factors.

The original self-care maintenance factor included two dimensions: health-promoting behaviors (items 1, 3, 8) and illness-related behaviors (items 2, 4, 5, 6). On the CC-SC-CII-Korean, however, self-care maintenance (items 1, 2, 3, 4, 5, 6, 8) was identified as a single dimension. This result differs from the results of a previous study conducted in China^24^. This finding may be a cultural phenomenon that reflects Korean treatment standards.

In Korean caregivers, getting enough sleep, being physically active, managing stress, and eating healthy foods tailored to the disease are considered health-related behaviors for self-care maintenance. In other words, health-promoting behaviors and illness-related behaviors are considered the same concept rather than strictly separated. Health-promoting behaviors directly related to diseases such as Items #5 and #6 (taking prescribed medications, keeping appointments) fall under illness-related behaviors and may seem not essential for health-promoting behaviors, but in this study, all of them were the same. This is similar to the results of a study that found that chronically ill patients recognize regular physical activity, healthy eating habits, regular medical care, and compliance with prescription drug intstructions as self-care behaviors^40,41^. In addition, lack of sleep can have adverse health effects on patients with chronic diseases, so 'sufficient sleep' is not only a health-promoting behavior, but also an illness-related behavior^42^.This is similar to improved disease-related clinical outcomes (blood pressure, cholesterol levels, etc.) in which stroke survivors conducted illness-related health promotion activities such as daily physical activity, proper sleep time, stress management, and cholesterol-controlled food^43^. More research is needed to achieve a deeper understanding of the implications of this result.

On the CC-SC-CII-Korean, the monitoring factor (items 9, 10, 11, 12, 13) was found to be the same as the original instrument. These items were for monitoring the patients' condition, treatment-related side effects, normal activities, and symptoms, as well as attention to mood changes in patients. The self-care monitoring of chronically ill patients involves not only systematic and continuous monitoring of the body, but also careful monitoring of psychological changes^7,12^. In Korean culture, patients rely on healthcare professionals' direction, so a difference between maintenance and monitoring is expected. Caregivers are taught by healthcare professionals to monitor the patient's health status, including weight, blood sugar, blood pressure, temperature, medication side effects, emotional changes, disease-related symptoms and disease-related health behaviors^44^.These results might reflect the benefits of education specifically on disease-related monitoring items for chronic disease patients and their family caregivers.

On the CC-SC-CII-Korean, management factor (items 14,15,16,17,18,19,20)—was found through CFA. The original instrument included two dimensions: autonomous behavior (items 14, 15, 16, 17, 20) and consulting behavior (items 18, 19). Autonomous behavior refers to behaviors that an individual chooses autonomously based on previous experiences when experiencing any symptoms. Consulting behavior represent behaviors that are encouraged by someone other than oneself, such as healthcare professionals^23^. This is somewhat different result from previous studies^23,24^. Our interpretation of the results of the one-dimensional model of this study sample is that in Korean healthcare culture, caregivers often consult and listen to advice from healthcare professionals. In addition, caregivers receive education in advance on necessary self-care actions, such as taking medications or changing diet and lifestyle, when a patient's symptoms change^43,45^. As a result, autonomous behavior and consulting behavior are closely related. However, these results are from a sample in our study and further research is needed to confirm this factor structure.

This instrument demonstrated construct validity, and we found significant and strong correlations between caregivers’ self-efficacy and three caregivers’ contributions to self-care. Our finding was supported by relevant theory^7^, and the results were similar to the previous studies^23,24^. We demonstrated that the CC-SC-CII-Korean was a reliable instrument with adequate internal consistency.

The core of this research was the translation and validation of instruments that have not hitherto been available for use with informal caregivers who support patients with self-management of chronic conditions. The CC-SC-CII-Korean provides a comprehensive assessment of the caregiver's contribution to self-care maintenance, monitoring, and management and helps caregivers detect potential gaps in these three areas. The CC-SC-CII-Korean could also assist health care providers and researchers in planning and implementing interventions for caregivers to improve self-care support for people with chronic conditions.

Self-care for chronic disease patients is a continuous process, and this instrument can both objectively monitor the caregiver's contribution to the patient's self-care and serve as a motivator for positive change.

The CC-SC-CII-Korean scale, which validity and reliability have been verified in this study, is expected to be useful in measuring the contribution of caregivers to self-care in chronic disease patients. However, this study has several limitations. First, the subjects of this study were recruited through convenience sampling among the family caregivers of chronic disease patients visiting university hospitals in Seoul and Yongin, which are relatively large cities in Korea. Therefore, caution is required when generalizing the results of this study, and in the future, repeated studies targeting family caregivers of chronic disease patients in various communities are required. Second, although the caregivers participating in this study included the possible range of family caregivers in Korean culture, it was not possible to identify differences in the contribution of caregivers to each patient's self-care according to caregiver’s characteristics. However, previous studies conducted in other cultures have suggested that caregivers may differ depending on age, population, patient comorbidity, and culture, and in the future, dyadic research can be conducted among patients and family care providers to investigate their interactions and the effects of managing the chronic disease^23,24^. In addition, the amount of self-care required varies according to the number of chronic diseases, specific diseases, and severity^46–48^, therefore, future studies should analyze various diseases characteristics. Third, while the caregivers in our sample consisted of family members, further studies with informal caregivers who had various relationships with patients, including friends, neighbors, and colleagues, are needed.

In conclusion, this study shows that the CC-SC-CII-Korean has good validity and reliability and can be used in clinical and research studies to evaluate the contributions of caregivers to self-care for chronically ill patients in Korea. This instrument can measure the contribution of caregivers to patients' self-care, which is significant given the annually increasing rate of chronic disease in Korea. This instrument also allows healthcare professionals to understand how well a caregiver contributes to better patient outcomes.

## Methods

### Research design and participants

This cross-sectional study was performed at chronic illness clinics in two university hospitals in South Korea. Between the two sites, a convenience sample of 230 adult caregivers of patients with chronic illness was enrolled. Considering that the sample size required to test psychometric properties of the instruments must be at least 10 times the number of items, which was 19^35^, at least 190 participants were required. Based on the literature, 230 people were targeted, considering a dropout rate of 10% for the questionnaire and a dropout rate of 5% due to insincere responses^35^. Therefore, as 230 people participated, the instrument was considered verified. The eligible criteria of informal caregivers were ≥ 18 years old, identified by the patient as the main informal caregiver (family or otherwise), and provided most informal care to the patient, able to read and write in Korean. Those with severe illiteracy (i.e., an inability to fully respond to the research questionnaire) were excluded.

All study participants gave written informed consent and were told of their right to withdrawthe study at any time. The study followed the principles delineated in the Declaration of Helsinki, was conducted with the approval of the Ethics Committee of Severance Hospital, and was performed according to the STROBE section guidelines.

### Instrument

#### Caregiver contribution self-care of chronic illness inventory (CC-SC-CII)

The Caregiver Contribution to Self-care of Chronic Illness Inventory (CC-SC-CII) is a 19-item instrument that acts as a caregiver version of the SC-CII. It specifically measures the contributions of caregivers to self-care maintenance, monitoring, and management of patients with chronic illness^23^. The maintenance subscale (7 items) measures the contributions of caregivers to the patient to do behaviors to maintain of health; the monitoring subscale (5 items) evaluates the behaviors that the caregivers contribute for patients monitoring signs and symptoms of condition; the management subscale (7 items) measures the contributions of caregivers to the patient managing the signs and symptoms of the condition. The CC-SC-CII asks caregivers to report their involvement in the self-care of patients. In instruments, a 5-point Likert scale is used for answers (from 1 “never” to 5 “always”). It uses a standardized score from 0 to 100, with higher scores reflecting greater caregiver contributions to self-care and 70 used as the threshold for appropriateness. The original psychometric properties of the CC-SC-CII support both construct validity in CFA (comparative fit index [CFI] ranging from 0.97 to 0.99 and root mean square error of approximation [RMSEA] ranging from 0.05 to 0.06 across the three scales) and support reliability (Cronbach's alpha ranging from 0.76 and 0.93; maintenance subscale 0.83; monitoring 0.93; management 0.76)^23^. The CC-SC-CII is an instrument whose original and Chinese versions have been verified to have compositional validity in three factors: caregiver contribution to self-care maintenance, caregiver contribution to self-care monitoring, and caregiver contribution to self-care management^24^.

### Study procedure

#### Translation/back-translation0

After receiving permission from the original author^23^, the CC-SC-CII was translated into Korean, following guidelines for transcultural adaptation^28^. The CC-SC-CII was translated into Korean by two independent Korean researchers who were fluent in English and had expertise in chronic illness. The Korean version of the CC-SC-CII was then translated back to English by a bilingual researcher. A committee was constituted of experts involved in research or work teams associated with chronic illness to ensure no loss of meaning appeared during the translation process and to establish parity with the original instrument. Seven experts were selected: two clinical nurses in chronic illness practice; two chronic illness research nurses, two professors from a college of nursing in Korea, and one professor from a college of nursing in the US.

After that, cognitive interviews using think-aloud techniques were conducted with the two chronic illness clinical nurses to verify that all items of the CC-SC-CII were easily and correctly understood.

#### Content validity verification

For the content validity of the CC-SC-CII-Korean instrument, two experts in the management of chronic illness patients, one nursing professor and one clinical practitioner, were chosen by the professional evaluation team to calculate suitability using item-content validity index (I-CVI) values, with 1 point for "not very suitable,” 2 points for "not suitable,” 3 points for "fit,” and 4 points for "very suitable^32^.” The selected items had 3 or 4 points 100% of the time^32^.

### Data collection

Data were collected between October 2021 and February 2022. When trained research assistants met with potentially eligible participants, they confirmed eligibility of caregivers and then elucidated the study purpose. After eligible caregivers who were interested in the study signed an informed consent form, they were asked to fill out questionnaires, including socioeconomic and clinical characteristics, CC-SC-CII-Korean, and Caregiver Self-Efficacy in Contributing to Patient Self-Care (CSE-CSC) scale^34^ on paper or online.

We added the ten-item CSE-CSC scale—derived from the Self-Care Self-Efficacy Scale (SC-SES)- to test a construct validity by hypothesizing that higher scores of the CC-SC-CII are associated with better caregiver efficacy in contributing to patient self-care^12^. The CSE-CSC measures caregiver efficacy in contributing to self-care maintenance, monitoring, and management for patients with chronic illness^34^. The CSE-CSC scale uses a 5-point Likert format, with responses from “not confident” to “very confident.” The CSE-CSC scores correspond to greater caregiver efficacy in contributing to self-care for patients with chronic illness^34^.

### Data analysis

The data were analyzed using SPSS version 25.0 and AMOS version 26.0 (IBM Corp. Armonk, NY, USA). The sociodemographic characteristics of participants were calculated as frequency, percentage, mean and standard deviation, and the normal distribution and internal consistency of the items were confirmed through item analysis of the instrument. The content validity test of the instrument was evaluated using a 4-point Likert scale from the expert group and calculated using the I-CVI.

The CC-SC-CII was developed according to the middle-range theory of self-care of chronic illnessand determined the three dimensions of the scale^23^. Confirmatory factor analysis was conducted to verify the construct validity, and the suitability of the model was evaluated by the absolute fit index, relative fit index, and parsimonious fit index. The absolute fitness index is an evaluation of the degree to which the collected data and the research model match for the suitability analysis of the question composition, and was adjusted by the 22 statistic (freedom, p-value), the normal chi-square (NC), and the approximate error average square (root mean error of application) (Adjust) (FIexA). The relative fitness index represents how accurately the researcher's structural equation model was measured by comparing it with a model without correlations between variables (Young model), and fit was confirmed by the standard fit index (NFI) and the comparative fit index (CFI). Regarding the criteria for each index to be evaluated as suitable, the smaller the 22 statistic, the larger the p-value, and if it is greater than 0.05, it is generally accepted that the suitability is high. However, the 22 statistic is sensitive to the number of samples, and as the size of the sample increases, the 22 statistic increases; thus, even if it is statistically significant, the suitability is not necessarily low. As an indicator used to compensate for this, it is considered appropriate if the NC, obtained by dividing the 22 statistic by the degrees of freedom, is 3.0 or less. RMSEA is very good if it is less than 0.05 and appropriate if it is in the range of 0.05–0.08. AGFI, NFI, and CFI are evaluated to be very good for values of 0.90 or higher, and can be evaluated as suitable in the range of 0.70–0.90. In addition, significance (critical ratio [C.R.]) confirmed the relationship between latent factors and constituent variables^49^. In order to secure the construct validity of the items, it is desirable that the standardized factor load (β) reach a minimum standard of 0.50 or more, the reliability of concept (CR) should be 0.70 or more and 0.95 or less, and the mean variance extraction index (AVE) should be 0.50 or more^33,49^.

The correlation between the CC-SC-CII score and the CSE-SSC scale score was investigated using Pearson correlation coefficients (r). Each caregiver’s contribution to patients’ self-care was expected to positively correlate to the theory and results of previous studies^7,12^.

Lastly, the reliability of the instrument was confirmed by item-total correlation and internal consistency between items using Cronbach's α values. When Cronbach's α value is between 0.70 and 0.80, the internal consistency reliability is good, and when it is 0.80 and 0.90, it is very high^35^.

### Ethics approval

The study was approved by the Institutional Ethical Committee at Severance Hospital (Approval No: 4-2021-0763).

### Consent to participate

Informed consent was obtained from all participants. Participants first agreed to participate in the study and then provided written informed consent. Participants’ personal information, such as names, initials, and family medical history, was kept confidential.

## Data Availability

The data generated and analyzed during the study are available from the corresponding author upon reasonable request.
